# A minimal kinase–phosphatase system and its lipid substrates self-organize into dynamic patterns

**DOI:** 10.64898/2026.07.27.740984

**Published:** 2026-07-29

**Authors:** Ting-Sung Hsieh, Beatrice Ramm, Qiwei Yu, Jonathon L. Yuly, James P. Pfister, Yinhao Wu, Junzhou Huang, Yigal Meir, Ned S. Wingreen, Vincent S. Tagliabracci

## Abstract

Competing lipid kinases and phosphatases are critical for organizing cellular membranes, but whether a minimal system can autonomously organize proteins and lipids into dynamic spatiotemporal patterns is unknown. Here, we report the in vitro reconstitution of the *Legionella* phosphatidylinositol (PI) 3-kinase MavQ and PI 3-phosphatase SidP. Together with their lipid substrates, PI and PI 3-phosphate (PI3P), these enzymes form a minimal self-organizing system that generates ATP-dependent spatiotemporal patterns, including traveling waves, on model membranes. These behaviors arise from MavQ’s cooperative membrane binding, SidP’s phosphatase activity, and the continual interconversion and redistribution of PI and PI3P within a conserved membrane pool. A reaction–diffusion model reproduces the observed dynamics and predicts that lipid conservation prevents patterns from propagating across membrane discontinuities, which we verify experimentally. Together, these findings establish enzymatic modification of membrane lipids as a distinct molecular strategy for biological pattern formation.

## Full Text Availability

The license terms selected by the author(s) for this preprint version do not permit archiving in PMC. The full text is available from the preprint server.

